# 1045. Safety of the Synthetic Saponin Adjuvant TQL1055: Preliminary Results from a First-in-humans Trial

**DOI:** 10.1093/ofid/ofab466.1239

**Published:** 2021-12-04

**Authors:** Sean R Bennett, Tyler Martin

**Affiliations:** 1 Adjuvance Technologies, Inc., Lincoln, Nebraska; 2 Adjuvance Technologies, Lincoln, Nebraska

## Abstract

**Background:**

Saponin adjuvants reliably enhance immune response to a variety of antigens, but their use is hindered by dose-limiting toxicities and supply constraints. TQL1055 is a semi-synthetic analog of the natural saponin adjuvant QS-21, rationally modified to improve tolerability and enable large-scale manufacturing. We previously showed that the combination of acellular pertussis vaccine (aP) and TQL1055 was well-tolerated and increased anti-pertussis toxin (PT) antibody responses in mice and rabbits, with a no observed adverse effect level (NOAEL) > 2000 mcg/dose.

**Methods:**

Here we report interim results from a Phase 1 first-in-humans dose-escalation study of TQL1055. Healthy adults 18 to 50 years of age were sequentially enrolled into 6 groups (n=12/group) and randomized 10:2 to receive one intramuscular dose of aP + TQL1055 or aP alone on Day 1. TQL1055 dose increased by group from 25 to 800 mcg (Figure 1). Local adverse events (AEs) (injection site pain, redness, swelling) and systemic AEs (fever, chills, headache, fatigue, myalgia, arthralgia, nausea, vomiting, diarrhea) were solicited through Day 8. Clinical laboratory panels (chemistry, hematology, coagulation) were performed on Days 1 (pre-dose), 8, and 29. Serious AEs were collected through Day 365. Antibodies to PT were assessed at all visits.

Figure 1. Study Design

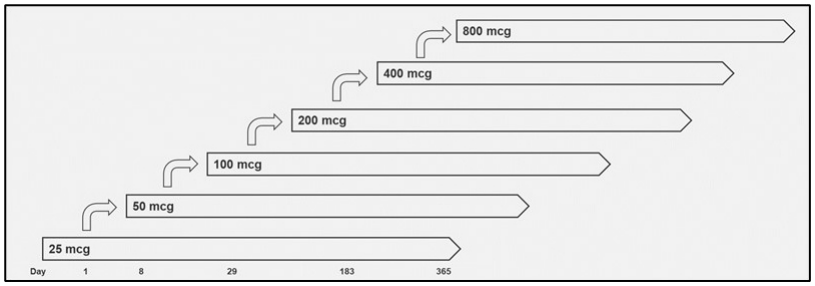

**Results:**

Blinded safety data from the first four groups (n=48) through Day 8 were analyzed, including 2 subjects/group receiving aP alone. All solicited AEs were mild or moderate (Figure 2). Local AEs, mainly injection site pain, occurred in 75% of subjects (mild 65%, moderate 10%). The incidence of total local AEs increased with TQL1055 dose, from 50% at 25 mcg to 92% at 200 mcg. The mean duration of local AEs was 1.8 days and also increased with TQL1055 dose, from 1.3 days at 25 mcg to 2.1 days at 200 mcg. Systemic AEs, mostly fatigue, headache, and nausea, occurred in 63% of subjects (mild 40%, moderate 23%), with no fevers. The mean duration of systemic AEs was 1.4 days, with no association with TQL1055 dose. No severe or serious adverse events were reported.

Figure 2. Solicited Adverse Events by Severity and TQL1055 Dose

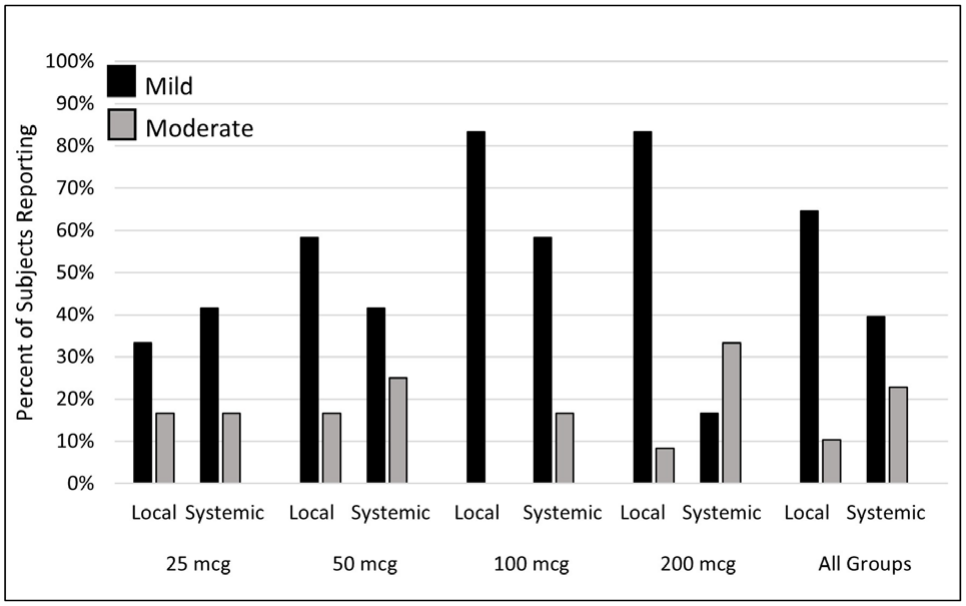

**Conclusion:**

In this early analysis, the safety profile of aP + TQL1055 appears similar to that of licensed aP vaccines, without severe or prolonged injection site pain. These data support further dose escalation and assessment of immunogenicity.

**Disclosures:**

**Sean R. Bennett, MD PhD**, **Adjuvance Technologies** (Employee) **Tyler Martin, MD**, **Adjuvance Technologies** (Employee, Shareholder)

